# Delivering a Quantitative Imaging Agenda

**DOI:** 10.3390/cancers15174219

**Published:** 2023-08-23

**Authors:** Nandita M. deSouza, Aad van der Lugt, Timothy J. Hall, Daniel Sullivan, Gudrun Zahlmann

**Affiliations:** 1 The Institute of Cancer Research, The Royal Marsden NHS Foundation Trust, London SW3 6JJ, UK; 2 Department of Radiology and Nuclear Medicine, Erasmus MC University Medical Centre, 3015 GD Rotterdam, The Netherlands; 3 Department of Medical Physics, University of Wisconsin, Madison, WI 53706, USA; 4 Department of Radiology, Duke University Medical Center, Durham, NC 27710, USA; 5 Independent Consultant for Quantitative Imaging Biomarkers Alliance (QIBA), Radiological Society of North America (RSNA), Oak Brook, IL 60523, USA

## 1. Introduction

In a digital image, each voxel contains quantitative information dependent on the technique used to generate the image. For example, the progression from plain film X-ray techniques to computed tomography meant that the information on tissue density could be extracted from each voxel and expressed in Hounsfield units. With functional imaging techniques such as positron emission tomography (PET), the signal detected directly reflects the number of emitted positrons generated by a radioisotope. Therefore, the standardised uptake value (SUV) from a region-of-interest quantitatively represents the extent of the metabolic process within this region. The modality of magnetic resonance imaging (MRI) is enormously versatile and allows the extraction of quantitative imaging biomarkers that reflect tissue relaxivity, perfusion, diffusion, and metabolism. In addition, it is now possible to obtain “radiomic” signatures from quantitative imaging data from multiple imaging modalities. Radiomics is the process of extracting statistical features in the data that are invisible to the naked eye [1]. Unfortunately, despite the enormous wealth of quantitative information available in the imaging data, quantitative data from clinical images are rarely exploited for clinical decisionmaking. Clinical trials in cancer, for example, still rely almost solely on Response Evaluation Criteria in Solid Tumours (RECIST) measurements [2], which, although simple to obtain, are merely unidirectional measurements of tumour burden and do not represent the complex architecture of tumours, nor the complex responses of tumours to an increasing array of targeted cancer therapies.

The reluctance to utilise quantitative imaging data in clinical trials and clinical practice stems largely from concerns about the variability in the measurements, thereby reducing confidence in the values themselves and in their repeatability and reproducibility [3]. This arises for a range of reasons, from variability in acquisition methodology, instrumentation performance, post-processing methods, and non-standard segmentation practices (i.e., defining the lesions or structures of interest in the image) to lack of reference standards. A roadmap was therefore agreed to by international consensus [4] that set out the necessary steps for taking an imaging biomarker from the discovery phase to clinical usage. There have been and are substantial international efforts to address these standardisation and implementation issues in Europe, North America, and other countries. The European Imaging Biomarkers Alliance (EIBALL) is a body of committed experts, both clinical and scientific, within the European Society of Radiology (ESR) that aims to promote standards for imaging biomarkers [5]. In North America, the Quantitative Imaging Biomarkers Alliance (QIBA), established 15 years ago, similarly has worked to develop and promulgate standards for quantitative imaging. These organisations work in partnership to identify clinically useful imaging biomarkers, document their state of development and usage, and develop methodologies and means for standardised acquisition, post-processing, validation, appropriate reference standards, and dissemination to the wider community.

## 2. Biomarker Inventory

The Biomarker Inventory is an EIBALL initiative designed to collate the status of available imaging biomarkers on the international consensus roadmap [4]. Biomarkers are categorised by organ system and by the data currently available to support their use, e.g., single-centre vs. multi-centre trials. The website is hosted by the ESR [6]. Each organsystem imaging biomarker panel was compiled through an extensive search of the literature by experts from organisations such as the European Organisation for Research and Treatment in Cancer (EORTC) and subsequently endorsed by the corresponding subspecialty society (e.g., European Society of Breast Imaging [EUSOBI], European Society of Gastrointestinal and Abdominal Radiology [ESGAR], and European Society of Paediatric Radiology [ESPR]). The list of presented biomarkers includes those that are considered established and those under development with the accompanying level of evidence. The limitations around usage are briefly indicated; these are notably around the lack of adequate standards in image acquisition, post-processing, and reference standards. The Biomarker Inventory is merely intended as a guide to researchers and clinicians on the range of available quantitative imaging biomarkers that can be exploited and the status of each biomarker in a particular organ system, rather than a guideline on recommended usage. As such, it involves regular updates from experts to ensure it is abreast of the current literature and standardisation efforts.

A key factor in the successful usage of the Biomarker Inventory is knowledge of its availability as a free resource. This has been driven by promotional events between the ESR and specialist societies through podcasts and social media. Awareness of the potential of available imaging biomarkers should also feed into wider standardisation efforts, such as the development of harmonised protocols, post-processing algorithms, and validated reference standards.

## 3. Standardised Processes/Protocols and Conformance Testing

The Radiological Society of North America (RSNA) established the Quantitative Imaging Biomarkers Alliance (QIBA), now in transition to a Quantitative Imaging Committee, to assist in transforming radiology from a qualitative to a more quantitative science and to improve patient care by accelerating the development and dissemination of new pharmacologic, biologic, and interventional diagnosis and treatment approaches. Individual imaging biomarker committees cover biomarkers derived from CT, MR, PET/SPECT, and US. A defined, coordinated process has been utilised to develop solutions to standardise biomarker development and promote their adoption [7].The work products, referred to as “Profiles”, standardise all the necessary steps to create an imaging biomarker that meets a performance claim (i.e., specified levels of accuracy and reproducibility). Formal metrology concepts are used. The profile generation process begins with stakeholders identifying sources of error and variation in the measurement of the selected quantitative imaging biomarker. Secondly, stakeholders specify potential hardware, software, and protocol solutions to reduce the errors and variability. A Profile that has been drafted and agreed on in a dedicated biomarker committee is sent out for public comment to the imaging biomarker community. All public comments are evaluated, and the Profile is adjusted towards consensus among imaging experts. Vendors and researchers implement and test these solutions to assess their feasibility and efficacy. To be clinically feasible, the consensus Profile should ideally be tested for conformance in at least three clinical imaging centres [8]. Each Profile includes a checklist that summarises all the necessary steps an imaging site needs to fulfil to achieve the described performance of the quantitative imaging biomarker.

It is anticipated that these validated solutions (Profiles) are then disseminated and implemented through vendor adoption, research integration, and clinical education. Ideally, a Profile claim can be confirmed in a clinical test–retest study.

Currently, more than 160 sites have tested their conformance to QIBA Profiles. This can be done via self-attestation by working with experts to provide all information as requested by the Profile checklist or via certification, in which experts compare the performance to a reference standard and confirm the results.

## 4. Reference Standards

Since true values of a quantitative imaging biomarker are rarely available in biological tissues, a reference standard material or object with independently known properties is needed to establish bias in the estimate of a biomarker. The use of tissue-mimicking phantoms (test objects whose properties are similar in significant ways to relevant tissue properties and whose properties can be independently validated) is well accepted in the medical imaging community to provide such a reference standard. Reference standards enable understanding the ability of an imaging system to estimate the true value of the biomarker in patients. This need for a “true value” in the definition of a quantitative imaging biomarker was extensively debated by QIBA’s Ground Truth Task Force [9]. Unfortunately, tissue-mimicking phantoms are not nearly as complex as tissue, which is a limiting factor in equating imaging system performance in phantoms to that in tissue. An excellent example of phantom use is for diffusion-weighted measurements in MRI where a verified phantom has been used to assess the repeatability of the apparent diffusion coefficient (ADC) biomarker prior to its quantification in prostate cancer [10], head and neck cancer [11], for cervical lymph node assessment [12], and for radiomic analysis of diffusion-weighted MRI in rectal cancer [13].

## 5. Summary

There is widespread recognition of the urgent need for standardising imaging biomarkers to increase their utility and uptake in order to deliver a reliable means of assessing the presence of disease and measure its response to treatment. Considerable international efforts from the imaging community are therefore addressing the process of identifying and harmonising imaging biomarker development. However, there is still much work to be done. To introduce imaging biomarkers into clinical practice, their initial uptake within clinical trials is essential. Working with trials organisations such as the EORTC, American College of Radiology Imaging Network (ACRIN), and National Cancer Research Institute (NCRI) in the UK are an essential step to introducing imaging biomarkers recognised within the Biomarker Inventory in a standardised way into relevant trials using validated Profiles. Triallists need to be educated on imaging biomarkers relevant to their clinical questions, and this may be best achieved through international meetings of the relevant societies both in radiology (e.g., European Congress of Radiology [ECR], Radiological Society of North America [RSNA], International Society of Magnetic Resonance in Medicine [ISMRM], European Association of Nuclear Medicine [EANM], Society of Nuclear Medicine [SNM]) and oncology (e.g., European Association for Cancer Research [EACR] and American Association of Clinical Oncology [ASCO]). Furthermore, raising awareness of the availability of these processes through funders both non-commercial and commercial will go a long way towards the incorporation of imaging biomarkers into the clinic. Ultimately, the use of properly generated and validated imaging biomarkers will greatly enhance clinical decision making in an era of personalised cancer treatments. We have therefore devoted this Special Issue of *Cancers* to “Quantitative Imaging and Digital Pathology in Clinical Cancer Research”. It focuses on bringing the use of validated imaging and pathology-derived biomarkers to a wide audience of end-users so that these biomarkers can be effectively incorporated into clinical trials and ultimately into clinical practice.
